# Microbiome insights into pediatric familial adenomatous polyposis

**DOI:** 10.1186/s13023-022-02569-2

**Published:** 2022-11-14

**Authors:** Thomas M. Attard, Seth Septer, Caitlin E. Lawson, Mark I. Attard, Sonny T. M. Lee, Shahid Umar

**Affiliations:** 1 Department of Gastroenterology, Children’s Mercy Hospital, 1MO2.37, 2401 Gilham Road, Kansas City, MO 64108 USA; 2 Department of Pediatric Gastroenterology, Children’s Hospital Colorado, Aurora, CO USA; 3 Division of Genetics, Children’s Mercy Hospital, Kansas City, MO USA; 4 Neonatal Unit, Aberdeen Maternity Hospital, Aberdeen, AB25 2ZL UK; 5 Division of Biology, Kansas State University, Manhattan, KS USA; 6 Department of Surgery, University of Kansas Medical Center, 3901 Rainbow Blvd, 4028 Wahl Hall East, Kansas City, KS 66160 USA

**Keywords:** Familial adenomatous polyposis, Colorectal cancer, Microbiome

## Abstract

**Background:**

Individuals with familial adenomatous polyposis (FAP) harbor numerous polyps with inevitable early progression to colon cancer. Complex microbiotic-tumor microenvironment perturbations suggest a dysbiotic relationship between polyp and microbiome. In this study, we performed comprehensive analyses of stool and tissue microbiome of pediatric FAP subjects and compared with unaffected cohabiting relatives through 16S V4 region amplicon sequencing and machine learning platforms.

**Results:**

Within our FAP and control patient population, Firmicutes and Bacteroidetes were the predominant phyla in the tissue and stool samples, while Proteobacteria dominated the polyp/non-polyp mucosa. A decline in *Faecalibacterium* in polyps contrasted with a decline in *Bacteroides* in the FAP stool. The alpha- and beta-diversity indices differed significantly within the polyp/non-polyp groups, with a concurrent shift towards lower diversity in polyps. In a limited 3-year longitudinal study, the relative abundance of Proteobacteria and Fusobacteria was higher in polyps compared to non-polyp and stool specimens over time. Through machine learning, we discovered that *Archaeon_enrichment_culture_clone_A13*, *Micrococcus_luteus,* and *Eubacterium_hallii* in stool and PL-11B10, S1-80, and Blastocatellaceae in tissues were significantly different between patients with and without polyps.

**Conclusions:**

Detection of certain bacterial concentrations within stool or biopsied polyps could serve as adjuncts to current screening modalities to help identify higher-risk patients.

**Supplementary Information:**

The online version contains supplementary material available at 10.1186/s13023-022-02569-2.

## Introduction

Colorectal cancer (CRC) is one of the most common malignancies worldwide and the third leading cause of cancer in the United States. Most cases of CRC are sporadic, but a clear familial predisposition is evident in up to 30% of individuals, with another 5% presenting in individuals with a cancer-predisposing syndrome [1].

Familial Adenomatous Polyposis (FAP) is a hereditary predisposition for the development of numerous colorectal adenomas that inevitably progress to colorectal cancer in early adulthood [2]. The underlying genetic abnormality resides in a mutation of the APC gene on chromosome 5q21. Affected individuals harbor germline mutation coding for a truncated protein and then acquire a second mutation that inactivates APC protein synthesis. This results in decreased intracellular Beta-catenin clearance and subsequent activation of the *Wnt*-wingless pathway that modulates cell polarity, migration, and proliferation. Although it is relatively uncommon (incidence; 1:8,000) the mechanisms driving tumorigenesis in FAP are virtually identical to the great majority of patients with sporadic colorectal cancer so that observations on microbiome and polyp interrelationships may have significant implications to the vast CRC burden in the general population.

Microbiota modulation of cellular physiology is thought to play a critical role both in health and disease, most notably in gastrointestinal illnesses including inflammatory bowel disease [3], including in children [4], and in cancer. The observed relationships include patterns of changes in specific bacterial subgroups or in measures of the diversity of the bacterial population in both healthy and diseased tissue or stool from affected individuals compared to healthy controls.

The discovery of a relationship between *Streptococcus bovis* endocarditis and CRC [5] paved the way for a trove of research ranging from in vitro, in-vivo, and subsequent human epidemiologic and metagenomics research [6]. Colorectal regional differences and more intimately biofilm-mediated specific interactions have demonstrated that the tumor microenvironment may be polyp [7] and site-specific. Pro- and anti-neoplastic bacterial species and phyla have emerged with sub-networks of co-occurring and co-excluding microbes at and around neoplastic sites [8]. The proposed mechanisms include bacterial oncoproteins, metabolite interaction with oncogenic pathways, and inflammatory mediators that indirectly modulate cell proliferation and apoptosis. The modulating effects of the microbiome may in part be associated with microenvironmental factors that influence adenoma progression and tumor development.

Our hypothesis was that mucosal-level microbiome influences and is influenced by polyp development in individuals with FAP. We postulate that differences we observe in the microbiome constitution of non-polyp and polyp mucosa reflects the microbiome characteristics that favor polyp growth, our aim therefore is to describe the permutations in microbiome between polyp and non-polyp mucosa in children with FAP with a secondary aim of comparing both with fecal derived microbiome.

Children with FAP represent a population at risk as well as a paradigm for the high likelihood of adenoma development with the potential to shed new insight on the mechanism of both syndromic and sporadic CRC tumorigenesis and metagenomics. Observations in this population may therefore have significant broader ramifications.

## Materials and methods

### Patient recruitment

The study was reviewed and approved, designated as no greater than minimal risk, by the institutional IRB of the participating site (Children’s Mercy Kansas City IRB# 13120420), subjects consented to participate or, if minor assented to participate in the study, with consent from their respective legal guardians prior to enrollment. All methods were performed in accordance with the relevant guidelines and regulations. Thirteen patients (7 Male) with FAP followed at the Multidisciplinary Clinic at Children's Mercy Hospital were recruited along with unaffected cohabiting family members designated as controls. Diagnosis of FAP was based on published, standard clinical criteria for children, genetic testing results were noted, when available. Neither subjects nor controls had received antibiotics up to two weeks pre-procedure, although one subject had received probiotics. Their mean age (SD) at the time of polyp/tissue sampling was 13.7 (3.31) years, and the cohort included two sibling pairs. Adenomatous polyps, defined as intraluminal projecting lesions measuring > 3–5 mm in diameter estimated by two experienced endoscopists (TMA, SS), were removed and submitted as polyp tissue along with a biopsy of normal-appearing (non-polyp) mucosa (natural and digital image enhanced endoscopy (IEE) distant to the polyp but within the same colorectal anatomic segment. A corresponding stool specimen was obtained within a week prior to the endoscopy and not from the time of pre-colonoscopy prep, was obtained and paired with a stool specimen in a prior determined unaffected sibling. Overall, 50 separate samples (25 tissue, 25 stool) were successfully submitted and processed (Table 1). In 4 samples, labelling was found to be incomplete; these samples were included only in the pooled analysis depending on the known parameters that were recorded (e.g., tissue undifferentiated; polyp or non-polyp mucosa, subject 3, 1st year, subject 5, 1st and 2nd year) (Table 2). Three subjects were referred to colectomy during the study period, although this was not an exclusion parameter.

**Table 1:** Subject and control demographic characteristics

Subject/control	Subject		Control	
	Gender	Age at procedure	Gender	Age
1	F	19	F	43
2	F	16	F	51
3	F	17	F	48
4	F	17	F	49
		19		51
5	M	10	M	13
		11		14
		12		15
6	M	9	F	29
7	F	12	M	45
8	M	6		
		7		
9	M	15		
		16		
10	M	14	F	50
		15		50
11	F	12	F	49
12	M	15	F	49
13	M	12	F	49

**Table 2:**
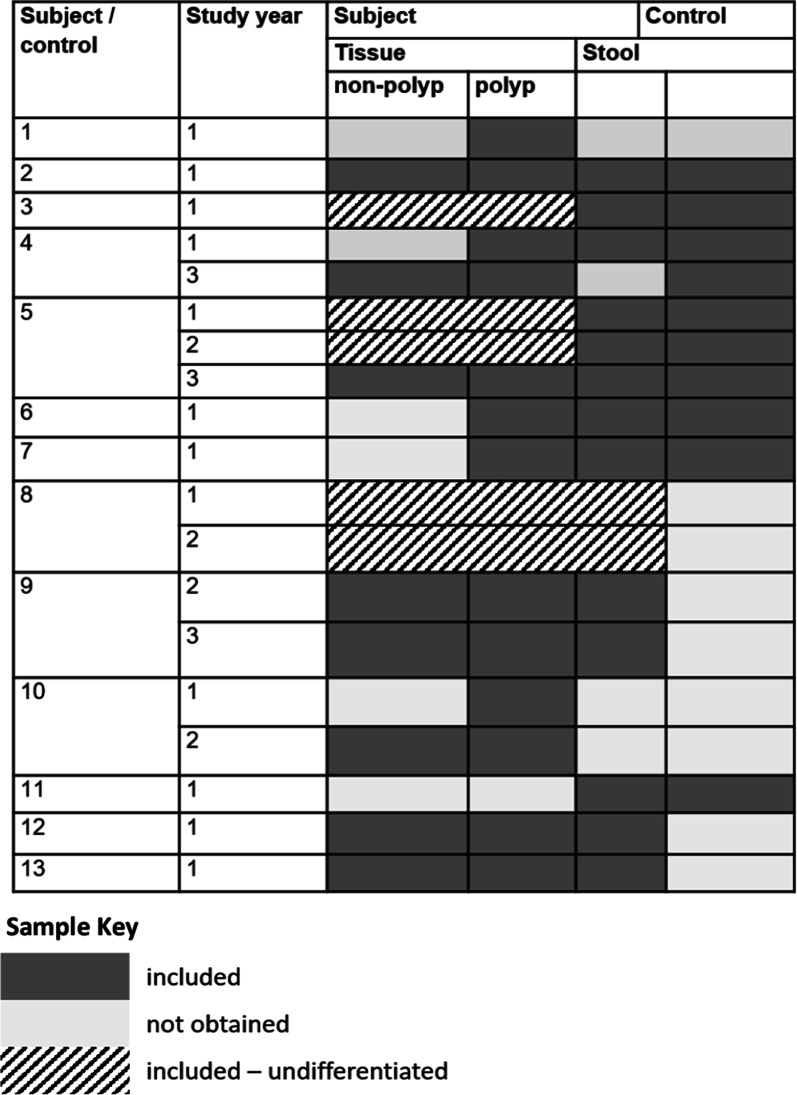
Samples

### Stool and tissue processing, bioinformatic and statistical analysis

Fecal specimens and adenomatous/normal tissues from FAP and non-FAP control subjects were collected and immediately frozen and stored at − 80 °C. DNA was extracted using a Power Soil Kit (MO Bio). The 16S V4 region was amplified using 515F/806R primers and sequenced using amplicon sequencing on IonS5^TM^XL to generate raw reads. Paired-end reads were assigned to samples based on their unique barcode and truncated by cutting off the barcode and primer sequences. We used Cutadapt [9] (V2.1, http://cutadapt.readthedocs.io/en/stable/) with parameters p-error-rate 0.1 to remove primers and adaptors from the sequences before performing downstream bioinformatic processes in QIIME2 v 2020.8.0. Briefly, we used QIIME2-wrapped DADA2 v1.14 (Callahan BJ, 2016) to remove chimeric and singleton sequences and join paired-end reads to provide the Operational Taxonomic Unit (OTU) table. The reads were compared with the Silva 132 (https://www.arb-silva.de/) [10] using pre-trained classifiers from QIIME2 data resources.

To study phylogenetic relationships of different OTUs, and the difference of the dominant species in different samples (groups), multiple sequence alignments were conducted using QIIME2. OTUs abundance information was normalized using a standard sequence number corresponding to the sample with the least sequences. Subsequent analyses of alpha diversity and beta diversity were all performed based on this output normalized data. Alpha diversity was applied in analyzing the complexity of species diversity for a sample through six indices, including Observed-species, Chao1, Shannon, Simpson, ACE, and Good-coverage. All these indices in our samples were calculated with QIIME2 and displayed with R software (Version 2.15.3). Beta diversity analysis was used to evaluate differences of samples in species complexity. Beta diversity on both weighted and unweighted unifrac were calculated by QIIME2. Principal Coordinate Analysis (PCoA) was performed to get the principal coordinates and for visualization of the complex, multidimensional data. A distance matrix of weighted or unweighted unifrac among samples obtained before was transformed to a new set of orthogonal axes, by which the maximum variation factor was demonstrated by the first principal coordinate, and the second maximum one by the second principal coordinate, etc. PCoA analysis was displayed by WGCNA package, stat packages, and ggplot2 package in R software (Version 2.15.3). Unweighted Pair-group Method with Arithmetic Means (UPGMA) Clustering was performed as a type of hierarchical clustering method to interpret the distance matrix using average linkage and was conducted by QIIME2. Statistical analysis was performed using PERMANOVA, *t *test, Wilcox and Tukey test in R Adonis package. We also use LEfSe and ANOSIM to determine if there were any statistical differences in the microbial composition between the FAP individuals and the control populations.

### Machine learning

We divided the cohort stool samples into training and validation sets (Additional file 3: Table S1). The training set consisted of six samples from controls with no polyps and seven samples from patients with polyposis. The validation set consisted of five samples from controls with no polyps and eight samples from patients with polyposis. We evaluated the training set with three different algorithms, representing simple and complex nonlinear methods: k-Nearest Neighbors (KNN), Support Vector Machines (SVM) with a linear kernel and Random Forest (RF). The caret package (Ver 6.0.86) was used in Rstudio Version 1.3.1073 using R Version 4.0.2. We chose the best algorithm on the basis of accuracy and kappa, after five-fold cross-validation with five repeats (Additional file 4: Table S2).

## Results

### Study population

The study cohort included 13 individuals under the age of 21 with clinically diagnosed FAP; the demographic characteristics of the study population are summarized in Table 1. Seven subjects were male, the mean (SD) age was 13.4 (3.7) years at the initial procedure. Table 2 summarizes the samples that were obtained and included in this study. Table 3 summarizes the subject genetic mutation testing, colonic polyp burden at the time of the sampling procedure and outcome defined by referral to colectomy within the follow-up period (2 years) from study completion. Most patients had an identified *APC* gene mutation, although had three tested negative on prior, early generation APC testing. Polyp burden varied across the cohort with no clear progression with time in those individuals sampled repeatedly. None of the study participants had polyps estimated larger than one cm in diameter; most (79%) were categorized < 5 mm. The initial (study) procedure was followed by colectomy in three patients within a 2-year follow-up observation period.

**Table 3:** Clinical characteristics of study subjects, colonoscopy findings, polyp burden and follow up (colectomy at 2 years)

Subject	Subject			Study year	Polyp burden			Outcome
	Gender	Age	APC mutation		Number	Size distribution/mm	Distribution	Colectomy (age)
1	F	19	POSITIVE	1	6–10	< 5	A,D	N
2	F	16	UNKNOWN	1	11–15	< 5	PC	N
3	F	17	4720delA	1	< 5	< 5	A,S,R	N
4	F	17	POSITIVE	1	< 5	5–10	D,R	N
		19		3	21–30	< 5	C,A,D,S,R	
5	M	10	3183del5	1	6–10	< 5	D,S,R	N
		11		2	5–10	< 5	C,A,D,S,R	
		12		3	< 5	< 5	C,A,D,S	
6	M	9	PV in promoter 1B region	1	6–10	< 5	D,S,R	N
7	F	12	453delA	1	6–10	< 5	T,D,S	N
8	M	6	Large deletion in the APC gene	1	16–20	< 5	PC	N
		7		2				
9	M	15	POSITIVE	2	31–50	< 5	PC	N
		16		3	31–50	< 5	A,T,D,S,R	
10	M	14	UNKNOWN	1	6–10	< 5	A,D,S,R	N
		15		2	16–20	< 5	S,R	
11	F	12	NEGATIVE	1				Y(13)
12	M	15	NEGATIVE	1	> 50	5–10	PC	Y(15)
13	M	12	NEGATIVE	1	11–15	< 5	PC	Y(14)

#### Differential regulation of gut microbiota in polyp versus non-polyp and fecal samples

**Fig. 1:**
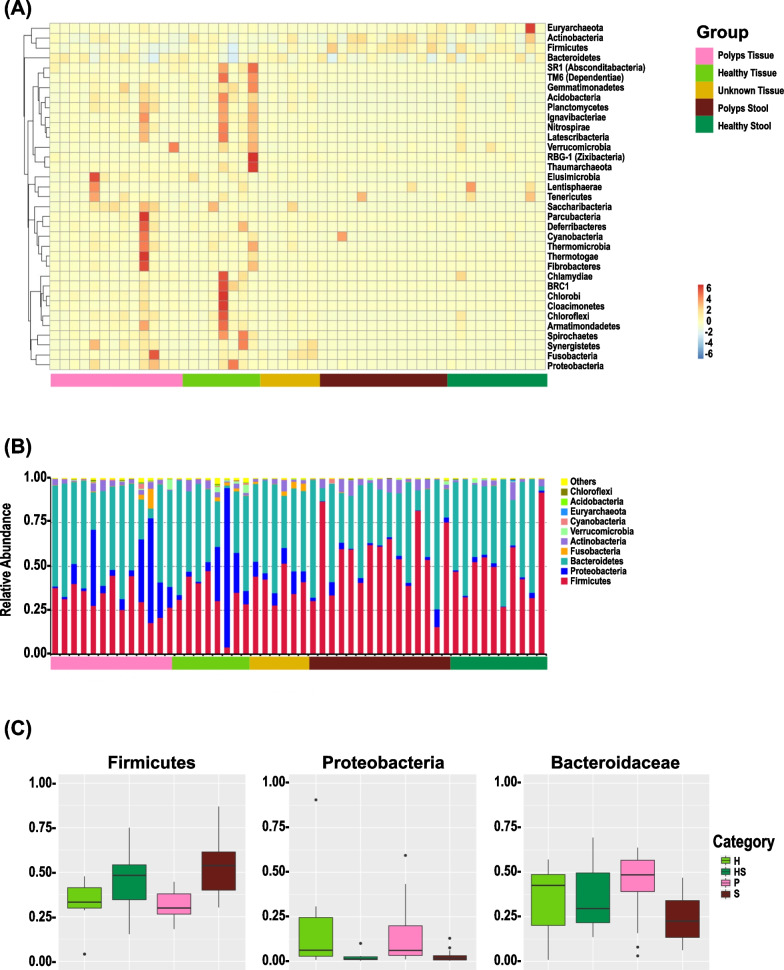
Changes in the relative abundance of top 10 phyla. Stool samples or biopsies were collected from healthy subjects or those with polyps every year for 3 years, followed by 16S gene sequencing. **A** Phylum relative abundance heatmap. The heatmaps displaying relative abundance distribution of dominant phyla among samples. **B** Relative abundance in specified groups. Histogram showing relative abundance in indicated groups. Arrows showing significant changes in indicated phyla. **C** Relative abundance in specified groups. Box plots showing the relative abundance of *Firmicutes*, *Bacteroidetes,* and *Proteobacteria* in specified groups. Wilcoxon rank-sum tests of the relative abundances, with *P* < 0.05 and detected in at least 70% of the samples, are shown

The gut microbiota profile was the single primary endpoint of the present study. On average, ~ 1450 bacterial OTUs were detected in stool or tissue samples combined (Additional file 1: Fig. S1A). The top 10 phyla in the different taxonomic ranks including Firmicutes *(*40.6 ± 12.1%), Bacteroidetes (43.6 ± 11.6%), Proteobacteria (10.3 ± 11.9%), Actinobacteria (3.2 ± 1.7%), Fusobacteria (0.9 ± 1.1%), Verrucomicrobia (0.6 ± 0.7%), Cyanobacteria (0.3 ± 0.4%), Euryarchaeota *(0.1* ± *0.1%)*, Acidobacteria (0.1 ± 0.1%) and Chloroflexi (0.1 ± 0.1%) formed the distribution histogram of relative abundance (Fig. 1A). The bacterial flora analysis showed that Firmicutes and Bacteroidetes were the predominant phyla of the tissue/stool microbiota in healthy or patients with polyps, respectively. In both the adenomatous polyp biopsy (P) and the adjacent non-polyp mucosa (H) however, the dominant phylum was Proteobacteria (P:19.5 ± 8.7%; H:20.1 ± 15.6%) compared to that found in stool samples from patients with polyps (S:1.7 ± 0.5%) or healthy subjects (HS:1.5 ± 1.1%) (Fig. 1A, B). Interestingly, the decline in relative abundance of Bacteroidaceae in the stool samples of FAP patients was more dramatic (Fig. 1B), suggesting that decreases in Bacteroidaceae relative abundance in stool may follow those recorded in adenomatous polyp or adjacent non-polyp mucosa.

When bacteria were agglomerated at the class, order, family, and genus levels, Gammaproteobacteria (7.0 ± 10.3%), Enterobacteriales (6.2 ± 9.6%), Enterobacteriaceae (6.2 ± 9.6%), and *E. coli-Shigella* (4.7 ± 7.6%) clusters predominantly represented the Proteobacteria phyla. Other significant changes recorded were a decline in the relative abundance of Bifidobacteriaceae belonging to Actinobacteria and Clostridia, Lachnospiraceae and Faecalibacterium belonging to the Firmicutes phyla (Fig. 1, Additional file 2: Figs. S1B–S1E). Interestingly, the presence of Fusobacteria was barely detected in the stools of either patient or healthy subjects (Fig. 1).

#### α-Diversity of gut microbiota in stool and tissue samples

**Fig. 2:**
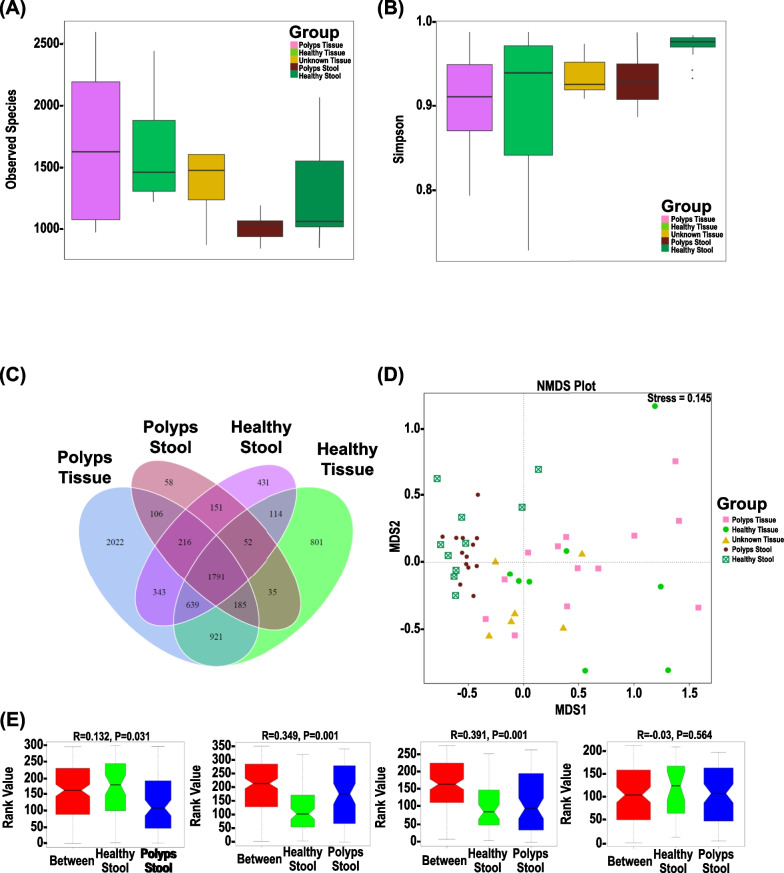
Variation analysis of alpha and beta diversity indices within and between groups. Alpha diversity indices of indicated samples showing observed OTUs (**A**), and Simpson (**B**) indices, respectively. **C**. Venn and Flower diagram. Venn diagrams showing shared and unique OTUs at 97% identity among the indicated groups. **D** Non-metric multidimensional scaling (NMDS). NMDS plot of tissues/stool bacterial community structure in the indicated groups. **E** Detailed ANOSIM analyses of most significant differences amongst groups. ANOSIM analysis was done for the comparison of β diversity. The *y*-axis represents the distance rank between samples, and the *x*-axis represents the results between both groups. R and *P* values are as indicated, with positive R values corresponding to significant differences supported by *P* values

Figure 2 shows the α-Diversity indices (observed species), which represent the richness and sequencing depth, and Simpson index that represents diversity within a sample. Overall, the bacterial diversity was significantly different among the samples (Fig. 2A, B). Adjacent non-polyp mucosa exhibited significant differences in microbiome diversity from polyps (P), adjacent non-polyp mucosa (H), stool (S) samples from subjects, and healthy controls (HS). (Fig. 2A, B). Venn diagrams representing the intersection of various microbiome taxonomic levels between datasets revealed 2022(P), 58(S), 431(HS), and 801(H) unique OTUs, while 1791 overlapping OTUs were shared by all the four groups (Fig. 2C). Predominant phyla belonging to these OTUs were Firmicutes, Cyanobacteria*,* and Proteobacteria, respectively.

### β-Diversity of gut microbiota in stool and tissue samples

**Fig. 3:**
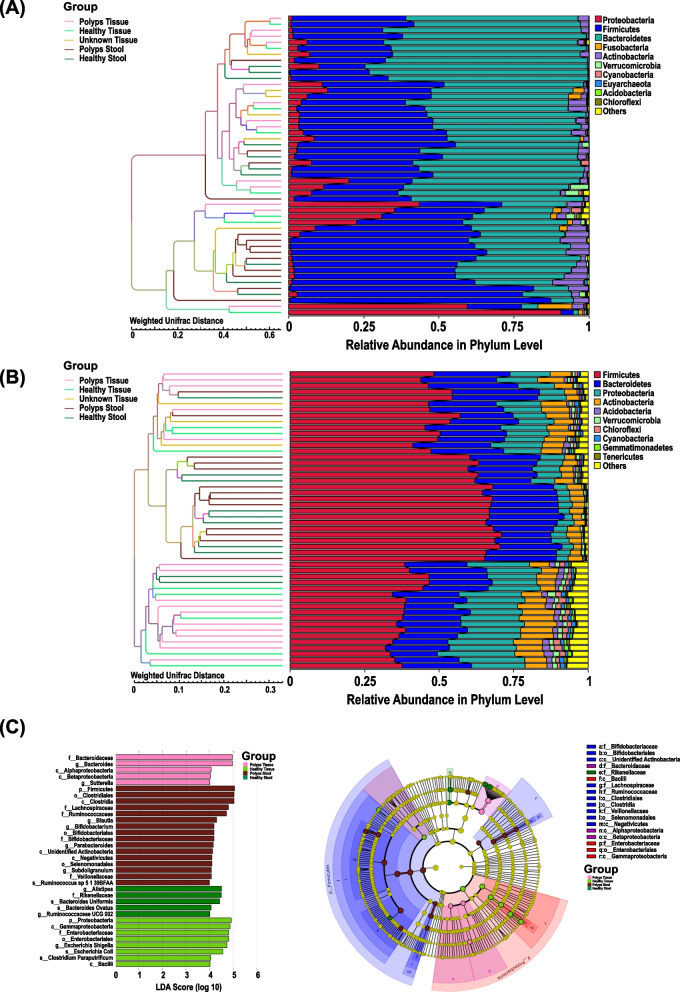
**A**, **B** Unweighted pair group method with arithmetic mean (UPGMA). UPGMA-clustering trees based on weighted (**A**) and unweighted (**B**) unifrac distances. **C** Linear discriminant analysis (LDA) Effect Size (LEfSe) analysis. A histogram of LDA scores was plotted to identify statistically significant biomarkers and to reveal the dominant microorganisms in the groups (left panel). Taxonomic comparison between adjacent non-polyp mucosa/stool samples. The taxonomic cladogram shows a comparison generated by GraPhlAn (Graphical Phylogenetic Analysis), representing high-quality, compact visualizations of microbial genomes and metagenomes (right panel). Colors distinguish between *Proteobacteria* (blue), *Firmicutes* (dark blue), *Bacteroidetes* (green), and *Actinobacteria* (red) phyla, while the intensity reflects the LDA score, an indicator of the effect sizes of the significant differences. The size of the nodes correlates with their relative and logarithmically scaled abundances. Taxa were both statistically significant (*P* < 0.05) and had an LDA Score > 4, considered a significant effect size

Non-metric multidimensional scaling (NMDS; stress 0.145) revealed significant alignment of P with H samples (Fig. 2D). NMDS for S or HS samples aligned together but clustered separately from either P or H samples, indicating clear site-specific differences in the microbial distribution in tissue and distant stool samples (Fig. 2D). Specifically, NMDS exhibited significant alignment of microbial communities in tissue or stool samples from FAP patients suggesting clearly that polyp and stool microbiomes are very distinct. Systematic group-wise comparison with a non-parametric permutation analysis of similarity (ANOSIM) confirmed that significant separation occurred between tissue and stool samples among groups at the level of R ranging between 0.35 (S vs. P; *p* = 0.001) to 0.391 (HS vs. P; *p* = 0.001) indicating that inter-group differences were greater than intra-group differences in microbial profile while ANOSIM revealed no significant separation within a patient between H or P group (Fig. 2E). Unweighted Pair-group Method with Arithmetic Mean (UPGMA) clustering algorithm on the weighted or unweighted UniFrac distances of samples further highlighted the segregation of bacterial communities in the tissue or stool samples (Fig. 3A, B).

We used LEfSe [11] and highlighted marked differences in the predominance of bacterial communities among groups (Fig. 3C). LEfSe plot displayed LDA scores of microbial taxa with significant differences in the tissue and stool samples not only within a patient but also between healthy subjects with the predominance of Alpha-/β-Proteobacteria, *E. coli-Shigella*, Fusobacteria and Bacteroidaceae (P), Gammaproteobacteria (H), and Clostridia and *Negativicutes* (S) at the class level and *Alistepis* genus belonging to the Bacteroidetes phyla (HS; Fig. 3C). These findings were corroborated by cladogram using the *export2graphlan* script provided with GraPhlAn that highlighted the differences in relative abundance among groups (Fig. 3C).

### Machine learning algorithms to identify bacterial communities

**Fig. 4:**
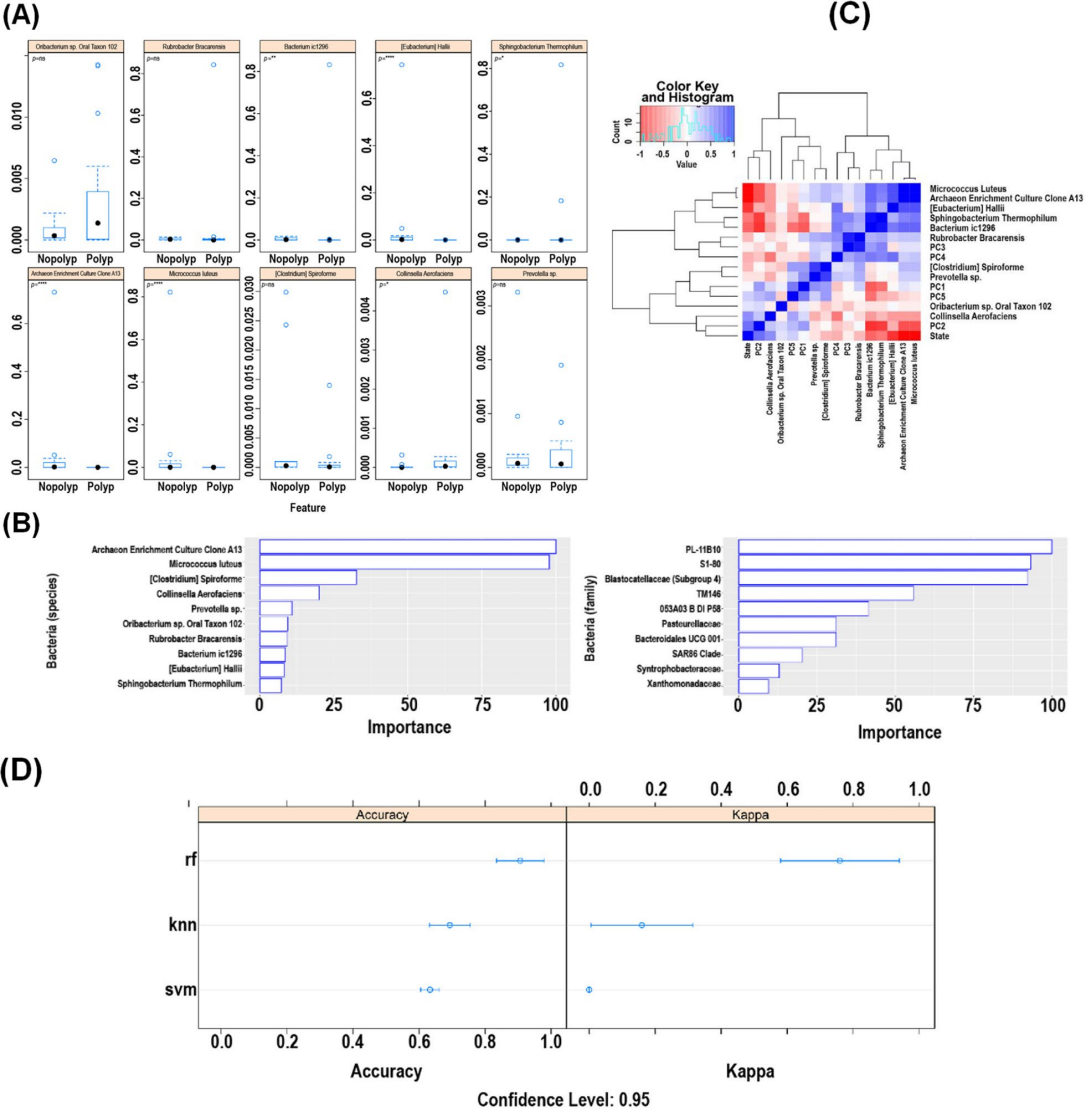
Machine learning findings. **A** The emerging ten most important bacteria in the machine learning models were compared between those affected with polyps and controls using Wilcoxon test (ns: *P* > 0.05; *: *P* ≤ 0.05; **: *P* ≤ 0.01; ***: *P* ≤ 0.001; ****: *P* ≤ 0.0001). **B** The emerging ten most important species and families according to percentage variable importance, in the stool (left) and tissue (right) machine learning models, respectively. **C** The correlation matrix showing distinguishing bacterial populations between patients with and without polyposis. **D** The RF model was used to predict non-polyp mucosa from polyp tissue in affected individuals

Amongst three machine learning models, the random forest (RF) model emerged, yielding the highest mean accuracy (0.81) and mean kappa values (0.64) on the training samples. This was applied to the validation samples to classify patients with FAP from controls. There were no misclassifications with a statistically significant accuracy metric. The top ten bacterial populations representing the most important variables in the RF model were assessed for correlations to patients with FAP (Fig. 4). We observed that *Archaeon enrichment culture clone A13*, *Micrococcus luteus,* and *Eubacterium hallii* distinguished stool from patients with and without polyposis (controls) (Fig. 4). We also observed that while the RF model was unable to predict non-polyp mucosa from polyp tissue in affected individuals at species and genus level, the accuracy was significantly higher at the family level. Bacteria from the three families—PL-11B10, S1-80, and Blastocatellaceae, were significantly different between patients with and without polyposis.

#### Differential regulation in longitudinal study

**Fig. 5:**
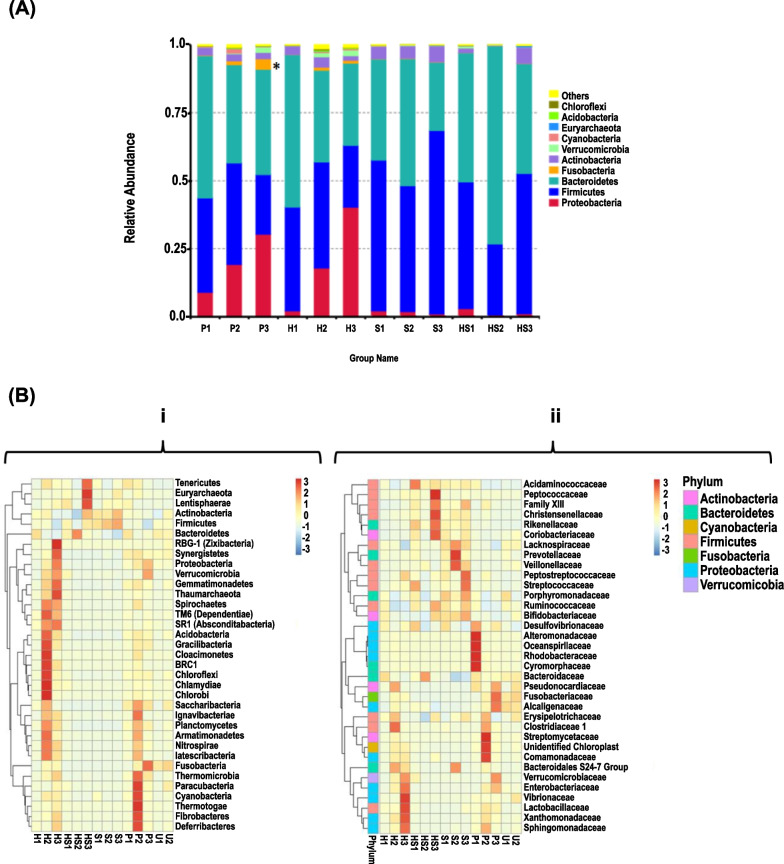
Bacterial communities in longitudinal study. Stool samples from patients: P1–P3, polyps in years 1–3; H1–H3, adjacent non-polyp tissue in years 1–3; S1–S3 and HS1–HS3, stool samples from subjects with polyps (S1–S3) and healthy subjects (HS1–HS3) in years 1–3. **A** Bacterial populations relative abundance in indicated groups. Arrows showing significant changes in indicated phyla. **B** Phylum and family abundance heatmaps. The heatmaps displaying a relative abundance of dominant 35 bacterial populations among indicated samples, representing the (i) phylum and (ii) family level in specified groups

A subset of the samples was analyzed longitudinally to determine if microbiome changes could be seen in multiple years of polyposis. We observed a gradual increase in Proteobacteria in polyps in years 1–3 (P1-P3) and in adjacent non-polyp mucosa (H1-H3) samples, respectively (Fig. 5A). In contrast, Proteobacteria levels in P1-P3 were negligible when measured in stool samples of either FAP patients or healthy subjects, clearly eliciting differences in the two sites (Fig. 5A). Firmicutes*,* on the other hand, declined from P1 to P3 or H1-H3, suggesting early dysbiosis in the development of polyps while the relative abundance remained steady in both S1-S3 or HS1-HS3 stool samples, respectively (Fig. 5A). At the phylum level, changes in Bacteroidetes were minimal in P1-P3. However, a generalized decrease in the *Bacteroides* genus in P1-P3 was accompanied by a similar trend in H1–H3 (Fig. 5B).

We also observed a generalized reduction in Firmicutes and Bacteroidetes*,* but increases in Proteobacteria phyla, especially Alpha- and Gammaproteobacteria*,* were seen in P1–P3 groups compared to either H1–H3, S1–S3 or HS1–HS3 groups, respectively suggesting that these changes may account for disease progression over time (Fig. 5B). Interestingly, changes in Lactobacillaceae in the P1–P3 samples were less dramatic with no gradual decline compared to H1–H3 samples wherein, we observed an increase in H3 samples (Fig. 5B). Relative abundance of Lactobacillaceae in the stool samples of FAP patients was less significant than either tissue polyps or adjacent non-polyp mucosa (Fig. 5Bii). Since *Fusobacterium* has been implicated in colon cancer progression, we observed a substantial increase in Fusobacteria*,* especially Fusobacteriaceae in the P1-P3 samples compared to H1–H3 wherein, the changes were less significant (Fig. 5B).

**Fig. 6:**
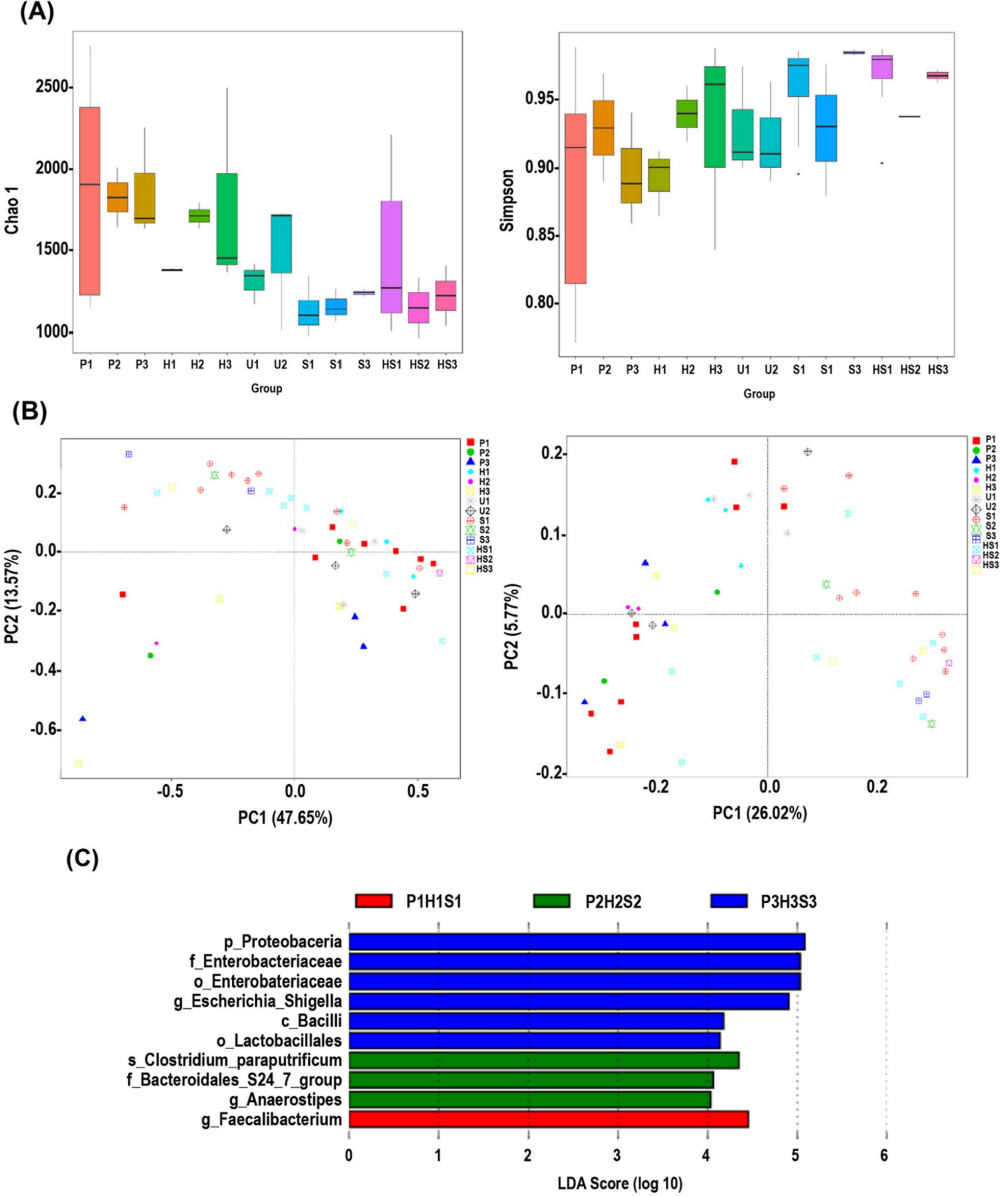
Bacterial communities’ diversity in longitudinal study. **A** Alpha diversity (Chao1) and Simpson indices. **B** Principal coordinates analysis (PCoA). Between-sample dissimilarities were measured by weighted (left panel) and unweighted (right panel) unifrac distances to assist the PCoA analysis. Each symbol represents a sample. **C** Linear discriminant analysis (LDA) Effect Size (LEfSe) analysis in longitudinal study. Taxonomic comparison between P1H1S1, P2H2S2, and P3H3S3 adjacent non-polyp mucosa/stool samples

Our results showed a decline in alpha diversity (Chao1) in the adenomatous tissues of year three FAP patients (P3) compared to year one (P1) (Fig. 6A). In contrast, adjacent non-polyp mucosa exhibited an upward trend with higher variability in H3 samples (Fig. 6A). However, when compared to P3 samples, a dramatic shift in Simpson alpha diversity from low (P3) to high (S3) with less variability was observed, suggesting distinct changes in the microbiota at these sites (Fig. 6A). Our results exhibited significant alignment of microbial communities in tissue or stool samples from FAP patients in year 1. In year 2, stool samples started to show separation from adenomatous tissues (Fig. 6B). In year 3, microbial communities from adenomatous tissues separated not only from the adjacent non-polyp mucosa but, more importantly, from the stool samples of FAP patients (Fig. 6B), reaffirming that polyp and stool microbiota are very distinct.

Our longitudinal analyses also indicated that there were several taxa with significant differences in years 1, 2, and 3. In year 1, the patients' tissue and stool were composed predominantly of *Faecalibacterium* (Fig. 6C). On the other hand, *Clostridium paraputrifcum, Bacteroidales S24_7,* and *Anaerostipes* were higher in relative abundance in year 2, while Proteobacteria, Enterobacteriaceae/ Enterobacteriales*, E. coli_Shigella,* Bacilli*, and* Lactobacillales dominated year three patients' samples (Fig. 6C). These results suggest that dysbiotic changes in these groups of bacteria may be integral to polyp evolution over time. Additional file 2: Fig. S2 is a schematic of our major findings.

Taken together, our findings indicate that both local (mucosa-related) and regional (stool-related) differences exist and that the longitudinal changes in the microbiota at these sites may facilitate polyp evolution over time.

## Discussion

Familial adenomatous polyposis (FAP) is inherited as an autosomal dominant trait, characterized by numerous adenomas in the colon and rectum that progress to colorectal cancer by the 4th decade of life [12]. Most patients with FAP harbor a germline mutation in the adenomatous polyposis coli (*APC*) gene. Evidence is emerging for the role of microbiota in FAP based on pre-clinical and clinical metagenomic studies along with pre-clinical studies using gnotobiotic hosts [7]. A detailed exploration of the spatio-temporal changes in the microbiome in the adenomatous tissues versus the stool samples, however, has not been achieved. We performed a high-throughput sequencing and bioinformatics analysis to characterize the tissue and stool microbiota of the FAP patients and compared them with healthy controls. The intent of the current study was to investigate if differences exist in microbiota composition between polyps and stool samples and whether the longitudinal changes in the microbiome may indicate susceptibility to developing colon cancer later in life.

In previous studies, differences in community composition between cancerous tissues and surrounding areas have led to a bacterial driver-passenger model for CRC [13, 14] observed changes in rectal mucosal bacterial communities of adenoma patients as well as in healthy controls and suggested that rectal mucosal bacterial composition may reflect the presence of adenoma-specific bacterial communities. We describe for the first time, differences in microbiota composition between polyps and adjacent non-polyp mucosa and re-affirm that the bacterial populations in feces and mucosa are distinct and may, in fact, differ in how they are enriched and/or distributed over a period of time. Firmicutes, Bacteroidetes, Actinobacteria*,* and Proteobacteria were the dominant phyla in healthy controls, similar to previous studies on gut bacteria [15]. A large decrease in Firmicutes, Bacteroidetes*,* and Actinobacteria coincided with the relative expansion of Proteobacteria in the adenomatous or synchronous tissues of FAP patients. These changes in Proteobacteria phyla in tissues/polyps in years one to three were much higher than those recorded in the stool. Proteobacteria have been shown to be enriched in adenoma compared with non-adenoma tissue from the same patient [16] and compared with tissue from healthy volunteers [17]. They are reported as a major phylum in colonic biofilms from FAP patients compared to healthy individuals [7]. Several larger studies reported significantly higher carriage or abundance of Proteobacteria in CRC [18, 19]. In our cohort, as with the observed increased abundance of Fusobacteria, these changes were not observed in patient or control stool samples suggesting distinct evolution of tissue and polyp microenvironment. Our observation of increased abundance of Fusobacteria in polyp tissue over time compared with healthy tissue or stool – where it was barely detected, is consistent with an increase in the prevalence and/or abundance of *Fusobacterium nucleatum* reported in the colorectal cancer tissue and fecal samples compared to individuals with colorectal polyps [20] and, may have important implications in CRC development in FAP. Fusobacteria is an adherent and invasive Gram-negative anaerobic bacterium usually residing in the oral cavity and associated with periodontal disease [21]. It is a potential risk factor for CRC progression [22, 23] and a higher abundance of *F. nucleatum* in CRC is associated with shorter survival [24]. Mechanistically, *Fusobacterium* induces the Wnt signaling through multiple mechanisms. Specifically, *F. nucleatum* interacts with TLR4 inducing β-catenin phosphorylation by PAK-1. The Wnt/β catenin pathway is also activated through *F. nucleatum*-produced FadA adhesin binding to E-cadherin, resulting in up-regulation of Annexin A1 [23, 25]. *Fusobacterium* modulates CRC proliferation through Toll-like receptor four signaling to MYD88, leading to activation of the Nuclear Factor-κB (NF-κB) [26], resulting in increased TNF-α, IL-6, IL-8, and miR-135b. We also observed mucosa-associated *E. coli*-*Shigella* cluster to be more prevalent in P1–P3 samples, which has been shown to encode cyclomodulin, vital for mutational changes in colon crypt cells [27]. Thus, increases in Fusobacteria and *E. coli*_*Shigella* clusters, especially in year three cohorts, further delves into the significance of mucosal dysbiosis in the evolution of CRC in FAP patients.

As a member of the *Clostridium leptum* group, *Faecalibacterium prausnitzii* could represent the beneficial commensal bacteria, and previous studies are consistent with the anti-inflammatory properties of this bacterium [28]. We observed a decline in *Faecalibacterium* belonging to Firmicutes phyla in the patients' tissue and stool polyps in years one to three (P1–P3) [29]. *Faecalibacterium* negatively correlates with inflammatory bowel disease activity and is relatively overexpressed in healthy tissue compared with CRC-polyps [30]. Mechanistically, this may be related to chemoprotective butyrate production, which correlates with dietary fiber intake [31]. Butyrate appears to induce tumor apoptosis through the expression of E-Cadherin [32]. *Faecalibacterium prausnitzii* spp., which promote short-chain fatty acids production (SCFA), including butyrate, are decreased in patients with advanced colorectal adenoma compared with controls [33]. Interestingly, in a murine model, a decline in other species including *Bacteroides uniformis* and *Bacteroides vulgatus* in cohorts encompassing polyps, synchronous tissues, and stools, correlated with disease progression [34]. *Bacteroides uniformis* utilizes the *O*-glycans covalently attached to mammalian mucin and serves as a mucin-degrader that predominantly colonizes the mucosal surfaces, thereby interacting with the host [35]. A *B. vulgatus* strain was shown to protect against *E. coli*-induced colitis in *IL-2*^*−/−*^ mice [38], while *IL-10*^*−/−*^ mice mono-associated with pig isolates of *B. vulgatus* had significantly reduced colitis-associated colon tumor multiplicity compared with conventional *IL-10*^*−/−*^ mice [36]. Thus, a decline in the levels of these species suggests that local microbiota disturbances may accompany disease progression.

When comparing the alpha diversity indices among groups using Student's *t*-test, we observed that both chao1 and Simpson indices declined in P3. Specifically, alpha diversity indices changed from low (P3) to high (S3) with less variability, suggesting that distinct changes in microbiome exist at these sites and that the development of adenomas/polyps may itself contribute towards microbiota imbalances. When we further delved into delineating the core microbiome at each site, we discovered ~ 1800 overlapping OTUs that were shared by the four groups in a Venn diagram regardless of whether it had high or low abundance. Yet, several unique OTUs were also present at each site. This combination of unique or overlapping core microbiomes may be integral to the development of CRC in FAP patients. The community structures of adenomatous/polyp tissues, when compared with stool samples, revealed hierarchical clustering upon PCoA analysis wherein, polyp and stool samples tended to cluster separately in FAP patients, particularly in year three (P3 vs. S3). These findings were further corroborated by ANOSIM data wherein, inter-group differences were significantly greater than intra-group variation in matched samples and by UPGMA clustering algorithm that clearly revealed segregation of bacterial communities in the tissue and stool samples. These site-specific alterations in the distribution of microbiota, whether causal or consequential, may dictate the kinetics of adenoma development as a prelude to CRC. In particular, we found that increased relative abundance of potential opportunistic pathogens such as Alpha/beta-Proteobacteria, *E. coli/Shigella*, Fusobacteria*,* etc., which contribute towards changes in intestinal homeostasis, might display robust inflammatory infiltration and directly or indirectly increase the risk of adenoma development.

We used machine learning to further provide insights into which bacterial populations are unique to patients with and without polyposis. The species that best discriminated between (stool from) subjects and controls were Archaea, *Micrococcus luteus,* and *Eubacterium hallii*. Euryarchaeota, the principal *Archaea* phylum in the intestinal microbiome, was one of the top 10 phyla in our combined OTU analysis. Euryarchaeota is highly diverse and includes methanogens, which in turn have been shown to be depleted in CRC [37] coincident with a progressive increase in halophilic spp. in stool from controls, adenoma then CRC. Furthermore, Archaea enrichment has been shown to relate to changes in the alpha-diversity observed in CRC [38]. Our observations suggest that perturbations in the Archaea subpopulation of the fecal microbiome may indeed modulate adenoma progression and may constitute useful biomarkers of syndromic adenomas. Another discriminant species in our ML analysis, *Eubacterium hallii*, distinguished stool from patients with and without polyposis (controls). *Eubacterium hallii* is recognized as a SCFA producing commensal and is decreased in diverse disease states, including inflammatory bowel disease and colorectal cancer [39].

*Eubacterium hallii* has been shown to detoxify carcinogenic heterocyclic aromatic amines present in processed meats [40]; its suppression, along with other SCFA producing species, has been implicated mechanistically in the relationship of high animal fat consumption in gut inflammatory and neoplastic processes [41]. Specifically, *E. hallii* has been proposed as a candidate next-generation probiotic [42].

The significance of *Micrcoccus luteus* is unclear; *M. luteus* is a well-described opportunistic pathogen usually in the context of immunocompromised hosts and has, to date, not been implicated in colorectal adenoma or cancer.

Thus, the realization that the microbiome modulates colorectal cancer risk introduces the possibility of altering the microbiome to change the risk of malignancy. Probiotics including *Lactobacillus* and *Bifidobacterium* have been shown to decrease adenoma formation in murine models of FAP [43, 44]. The mechanisms underlying the antitumor effects of probiotics include modulation of inflammatory pathways including NF-κB and downregulation of β-catenin; mechanisms that our observations suggest are active in the progression of adenomatous polyp evolution in our population. Specifically, for example, *Lactobacillus* supplementation has been shown to increase *Roseburia* in the *ApcMin/* + mouse model reversing one of the characteristics of polyp evolution observed in our study [44]. The polyp-microbial interrelationships we observed in patients with FAP may be interpreted in the context of potential pre- and probiotic therapy, including a putative role for *E. hallii*.

Our study has several limitations. Given the rarity of the underlying diagnosis of pediatric pre-colectomy FAP, accrual of a robust cohort was difficult. Maintaining regular surveillance and sampling as well as identifying and recruiting a suitable control was challenging. Accordingly, we could not obtain all planned samples over the three-year study period in any of our patients. This does not detract, however, from the validity of observations between different sample groups during the same year. Recent genetic testing was not available in all subjects, this could account for some of the subjects having been tested several years ago, with less sensitive testing modalities, reported as APC mutation negative. Our choice of controls inherently presented a challenge insofar as cohabiting, unaffected, same-gender individuals would be preferred but was unfeasible in view of siblings, if any, in most instances being either younger and untested, or older and not cohabiting, we therefore consciously chose cohabitation, being the stronger determinant of similarity [45], and therefore necessarily the study design favored the inclusion of unaffected parents skewing the mean age of the control population. Ultimately, we recognize that any choice of control poses potential pitfalls as many factors [46, 47] influence the intestinal microbiome and therefore any realistic control cannot be perfectly matched. This may have a bearing on the observed differences between subject and control stool. Similarly with the challenge of control samples, we understand the small sample sizes might limit our interpretation of our results. However, we minimized the error in our study due to effect size, by (1) adopting robust comparison of between and within sample groups for PERMANOVA analyses [48]; (2) ensuring the samples satisfied the t-test assumptions [49]; (3) ensuring the LEfSe analyses are performed by ranking based on magnitude of variation and not statistical significance [11].

As implied above, the principal limitation of our study is the attrition in samples in years two and 3. We have therefore adopted a strategy to separately analyze year one where the bulk of the samples are, and then we examined the findings from longitudinal analyses of the small subset that have longitudinal samples. Our present work provided the first step into the understanding of the microbiome shifts due to FAP, and future work could be performed with more samples to provide further insights.

Our study further highlights the limitation of stool-sampling strategies in defining the polyp microenvironment in adenomatous polyposis and by extrapolation sporadic polyp. Although some interrelatedness between polyp, healthy mucosa, and stool microbiome was apparent, it is exceedingly challenging to correlate polyp microenvironment changes from stool samples. Future research may focus on whether the detection of certain bacterial concentrations within stool or biopsied polyps could serve as adjuncts to current screening modalities to help identify higher-risk patients.

## Conclusions

We conclude that in children with FAP, the adenomas represent a devolution toward a more simplified bacterial community with key components having established pro-oncogenic characteristics when compared with uninvolved mucosa and stool and that the longitudinal changes in the microbiome at these sites even though limited in sample sizes, may facilitate polyp evolution over time.

## Supplementary Information

**Additional file 1: Fig. S1** A. Construction of OTUs and effective reads data. ~1450 bacterial OTUs were detected in stool or tissue samples combined. The y1-axis represents the number of reads. Red and blue bars represent the number of effective and annotated reads, respectively. We also detected some "Unique Reads" (orange bars) with a frequency of 1 that only occurs in one sample. The y2-axis titled "OTUs Number" represents the number of OTUs displayed as purple bars to identify the numbers of OTUs in different samples. B-E. Relative abundance of bacterial kingdom. Agglomeration of bacteria at the class, order, family, and genus levels. The y1-axis represents the number of reads. Red and blue bars represent the number of effective and annotated reads, respectively. We also detected some "Unique Reads" (orange bars) with a frequency of 1 that only occurs in one sample. The y2-axis titled "OTUs Number" represents the number of OTUs displayed as purple bars to identify the numbers of OTUs in different samples.

**Additional file 2: Fig. S2** Schematic of our major findings. The microbiome from fecal specimens with that obtained from normal mucosa in children with FAP and from fecal specimens in healthy controls was compared in addition to biopsies from an adenoma to synchronous normal mucosa. Observations including significant preferential expression of probiotic candidate bacteria in control stool vs. decreased potentially protective bacterial subtypes in polyp compared with normal mucosa were made along with a reduction in alpha- and beta-diversities in polyp compared with apparently normal mucosa. Machine learning further distinguished bacterial populations between patients with and without polyposis. Changes are indicated through up or down arrows, respectively. Partially created in BioRender.

**Additional file 3.** The training and validation sets used in machine learning methods.

**Additional file 4.** Accuracy metrics for the machine learning models.

## Data Availability

All datasets generated during the current study are available from the following hyperlink: https://www.ncbi.nlm.nih.gov/bioproject/PRJNA729439. The NCBI BioProject ID is PRJNA729439.

## Abbreviations

FAP: Familial adenomatous polyposis
CRC: Colorectal cancer
OUT: Operational taxonomic unit
NMDS: Non-metric multidimensional scaling
ANOSIM: Analysis of similarity
UPGMA: Unweighted pair group method with arithmetic mean
LefSe: Linear discriminant analysis effect size
RF: Random Forest
