# Supplementary figures and images for: Microbiome insights into pediatric familial adenomatous polyposis
Source: Orphanet J Rare Dis. 2022 Nov 14;17:416. doi: 10.1186/s13023-022-02569-2 (PMC9664625; doi:10.1186/s13023-022-02569-2)

A.

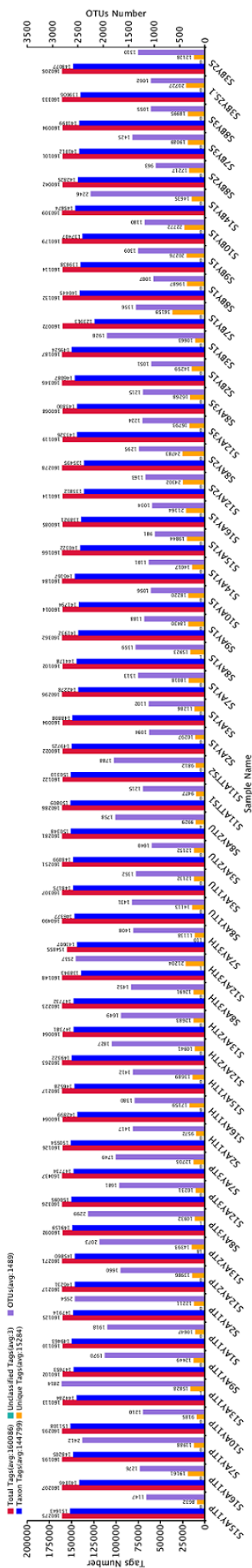

B. Class

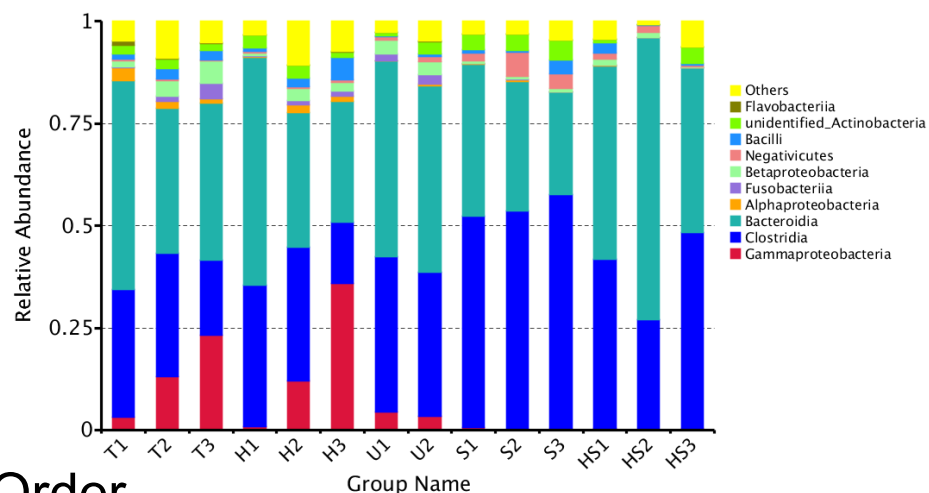

C. Order

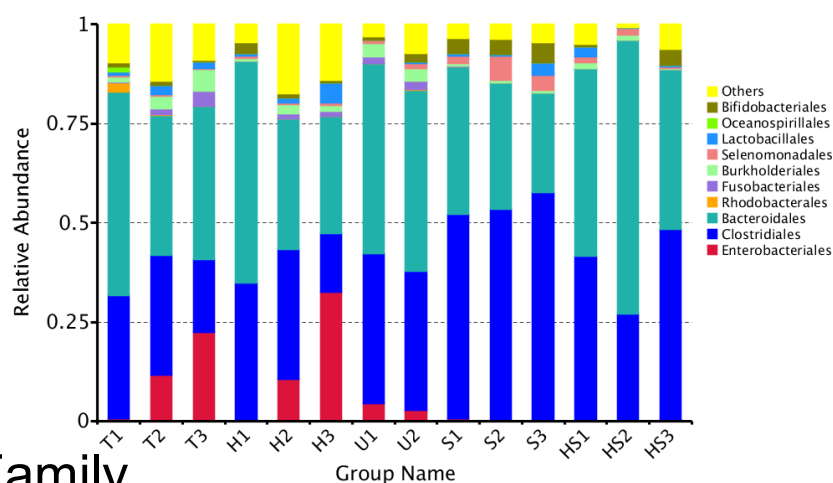

D. Family

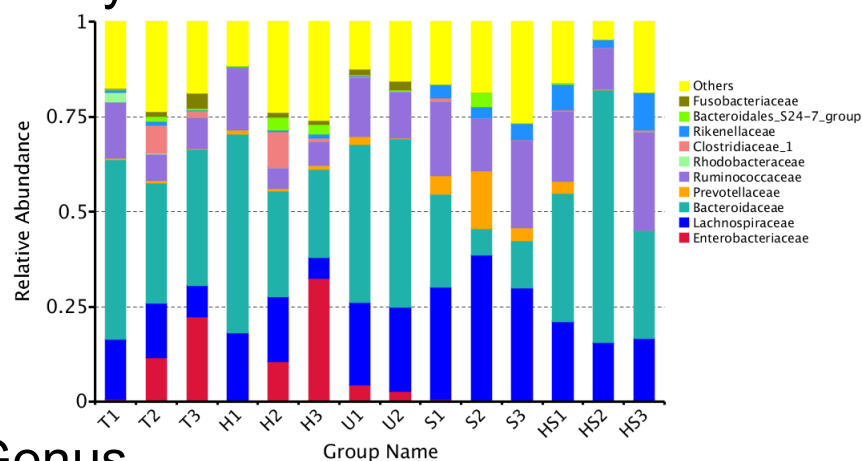

E. Genus

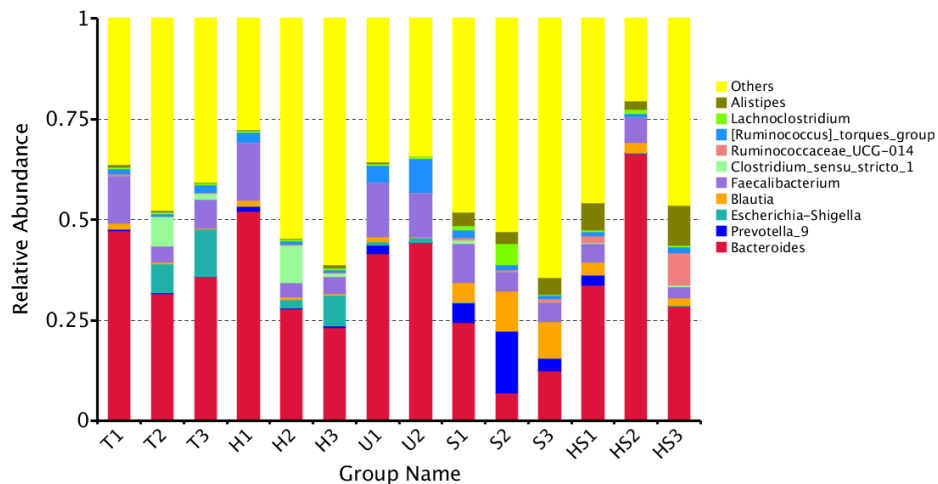

Supplement: Supplementary file 1 — Additional file 1: Fig. S1 A. Construction of OTUs and effective reads data. ~1450 bacterial OTUs were detected in stool or tissue samples combined. The y1-axis represents the number of reads. Red and blue bars represent the number of effective and annotated reads, respectively. We also detected some "Unique Reads" (orange bars) with a frequency of 1 that only occurs in one sample. The y2-axis titled "OTUs Number" represents the number of OTUs displayed as purple bars to identify the numbers of OTUs in different samples. B-E. Relative abundance of bacterial kingdom. Agglomeration of bacteria at the class, order, family, and genus levels. The y1-axis represents the number of reads. Red and blue bars represent the number of effective and annotated reads, respectively. We also detected some "Unique Reads" (orange bars) with a frequency of 1 that only occurs in one sample. The y2-axis titled "OTUs Number" represents the number of OTUs displayed as purple bars to identify the numbers of OTUs in different samples. [file 13023_2022_2569_MOESM1_ESM.pdf]

# Supplementary Figure 2

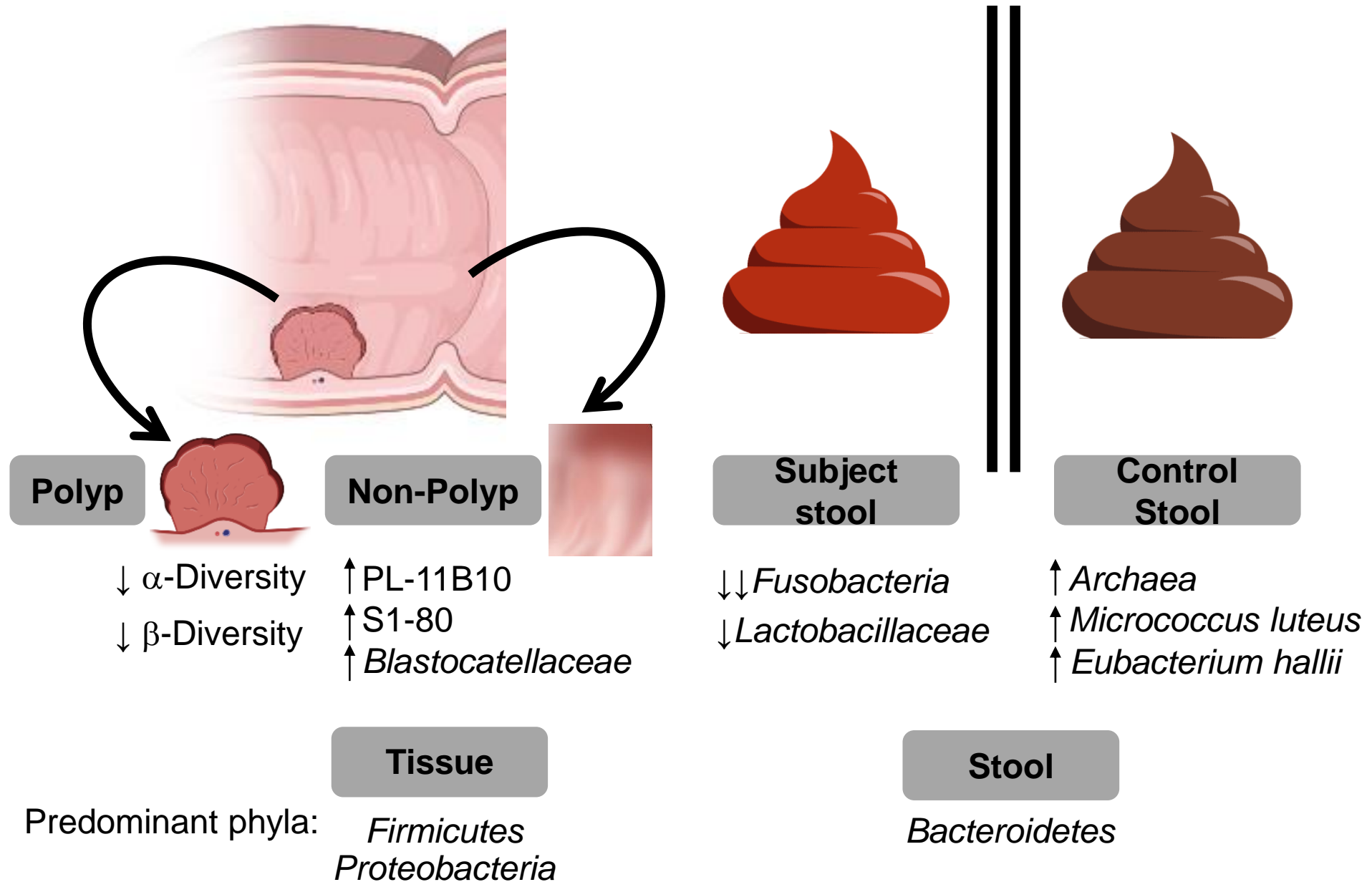

Supplement: Supplementary file 2 — Additional file 2: Fig. S2 Schematic of our major findings. The microbiome from fecal specimens with that obtained from normal mucosa in children with FAP and from fecal specimens in healthy controls was compared in addition to biopsies from an adenoma to synchronous normal mucosa. Observations including significant preferential expression of probiotic candidate bacteria in control stool vs. decreased potentially protective bacterial subtypes in polyp compared with normal mucosa were made along with a reduction in alpha- and beta-diversities in polyp compared with apparently normal mucosa. Machine learning further distinguished bacterial populations between patients with and without polyposis. Changes are indicated through up or down arrows, respectively. Partially created in BioRender. [file 13023_2022_2569_MOESM2_ESM.pdf]
