# Supplementary material for: Microbiome insights into pediatric familial adenomatous polyposis
Source: Orphanet J Rare Dis. 2022 Nov 14;17:416. doi: 10.1186/s13023-022-02569-2 (PMC9664625; doi:10.1186/s13023-022-02569-2)
Supplement: Supplementary file 3 — Additional file 3. The training and validation sets used in machine learning methods. [file 13023_2022_2569_MOESM3_ESM.docx]

**Supplementary Table S1: The training and validation sets used in machine learning methods.** Pseudonymised sample identification data were divided into training and validation sets.

| **Set** | **Stool Samples** | | **Tissue Samples** | |
| --- | --- | --- | --- | --- |
|  | **Polyps** | **No Polyps** | **Polyps** | **No Polyps** |
| Training | S2AY1S  S3AY1S  S7AY1S  S8AY1S  S9AY1S  S8AY2S  S8AY3S | S2BY1S  S3BY1S  S7BY1S  S9BY1S  S7BY3S  S3BY2S | S9AY1TP  S10AY1TP  S12AY2TP  S13AY1TP  S13AY2TP  S15AY1TP  S16AY1TP  S12AY3TP  S7AY1TP | S16AY1TH  S12AY3TH  S13AY2TH  S15AY1TH  S12AY2TH |
|  |  |  |  |  |
| Validation | S11ATTS1  S11ATTS2  S10AY1S  S14AY1S  S15AY1S  S16AY1S  S12AY2S  S12AY3S | S8BY1S (13 years old)  S10BY1S  S14BY1S  S8BY2S (14 years old)  S8BY3S (15 years old) | S1AY1TP  S2AY1TP  S7AY3TP  S8AY3TP | S2AY1TH  S7AY3TH  S8AY3TH |
