# Supplementary material for: Microbiome insights into pediatric familial adenomatous polyposis
Source: Orphanet J Rare Dis. 2022 Nov 14;17:416. doi: 10.1186/s13023-022-02569-2 (PMC9664625; doi:10.1186/s13023-022-02569-2)
Supplement: Supplementary file 4 — Additional file 4. Accuracy metrics for the machine learning models. [file 13023_2022_2569_MOESM4_ESM.docx]

**Supplementary Table S2: Accuracy metrics for the machine learning models.** (**A**) Models applied in the stool sample analysis. (**B**) Models applied in the tissue sample analysis.

(**A**)

| **Stool** | | |
| --- | --- | --- |
| **Model** | **Mean Accuracy** | **Mean Kappa** |
| KNN | 0.53 | 0.00 |
| SVM | 0.6 | 0.17 |
| RF | 0.81 | 0.64 |

(**B**)

| **Tissue** | | |
| --- | --- | --- |
| **Model** | **Mean Accuracy** | **Mean Kappa** |
| KNN | 0.69 | 0.16 |
| SVM | 0.63 | 0 |
| RF | 0.91 | 0.76 |
